# More Than Fifty Percent of the World Population Will Be Myopic by 2050

**DOI:** 10.14744/bej.2021.27146

**Published:** 2021-12-17

**Authors:** Ali Nouraeinejad

**Affiliations:** Department of Ophthalmology, Tehran University of Medical Sciences, Tehran, Iran

Dear Editor,

Myopia is the most common source of distance vision impairment in the world (1-5). It is creating an alarming global epidemic issue affecting the quality of life and economic health of individuals making socio-economic problem (1-5). Myopia also raises the risk of serious ocular diseases such as myopic macular degeneration (MMD), retinal damage, glaucoma, and cataract (1). In this regard, MMD has been reported to be the most common etiological factor of visual impairment in patients with myopia (1). MMD shows manifestations of “diffuse chorioretinal atrophy,” “patchy chorioretinal atrophy,” “lacquer cracks,” “choroidal neovascularization,” and “related macular atrophy” in the presence of high myopia (1).

There are financial burden and trouble with both uncorrected myopia and the cost of treatment of myopia with optical and non-optical modalities (1, 2). Frequent and long-term management of myopia by the eye-care practitioner are also added to the overall load of myopia (1-4). The earlier the onset of myopia, the greater is this load which may lengthen over many years and probably over the lifetime of the myopic person (1-4). A decrease in productivity, loss of quality of life, and independence among those affected will impact a significant health and socio-economic burden for the society as a whole (1-4).

Based on the prevalence data and the corresponding population figures worldwide, it can be revealed that myopia and high myopia will involve 52% (4949 million) and 10.0% (925 million), correspondingly, of the world’s population by 2050 (1, 2, 5). Therefore, the global prevalence of myopia is predicted to increase from 27% of the world’s population in 2010 to 52% by 2050 (1, 2, 5).

As the number of people with myopia increases, age of onset of myopia decreases (1, 2). This is of great concern as the earlier the onset, the more myopic the individual will become later in life (1). In this context, the prevalence of myopia has been shown to be more than two-fold over the past 50 years in white British children aged between 10 and 16 years old in the United Kingdom (6). They are also being myopic at a younger age (6).

As a result, myopia warrants national and international collaborative efforts as the cost and public health consequence are massive and frequently underrated in the trends (2).

## Disclosures

**Peer-review:** Externally peer-reviewed.

**Conflict of Interest:** None declared.
